# Seasonal dynamics of female bill colouration: an observation in captive House Sparrows (*Passer domesticus*)

**DOI:** 10.1007/s10336-025-02326-9

**Published:** 2025-09-26

**Authors:** Anastasia Caluja, Sebastian G. Vetter-Lang, Lisamarie Lehner, Katharina Mahr

**Affiliations:** 1 Department of Interdisciplinary Life Sciences, Konrad Lorenz Institute of Ethology, University of Veterinary Medicine Vienna, Vienna, Austria; 2 Centre for Food Science and Veterinary Public Health, Clinical Department of Farm Animals and Food System Science, University of Veterinary Medicine Vienna, Vienna, Austria; 3 Department of Botany and Biodiversity Research, Faculty of Life Sciences, University of Vienna, Vienna, Austria

**Keywords:** Female ornaments, House Sparrow, Bill colouration, Sexual selection

## Abstract

Bill colouration is a highly variable trait with the potential to rapidly change over time. We observed strong interindividual and seasonal variation in the bill colour of captive female House Sparrows (*Passer domesticus*) and describe two colour traits by using a simple categorization: i) the presence or absence of yellow markings on the upper mandible and ii) whether the female bill is light horn-coloured or dark brown. In spring, females display either light horn-coloured or dark, almost black, bills and half of the birds have yellow markings on the upper mandible. However, this changes markedly when the birds start breeding. In our observation, after the first brood, the proportion of individuals with yellow markings was significantly lower and in none of the females that raised offspring this trait was present. However, during the non-reproductive period in autumn, the yellow pigmentation of the upper mandible became visible in all individuals. Furthermore, the proportion of females with light bills increased from pre-breeding to the non-reproductive stage. The distinct differences in bill colouration together with the seasonal changes may indicate a potential signalling function and might serve in sexual selection.

## Abstract (de)

**Saisonale Dynamik der Schnabelfärbung bei Weibchen: eine Beobachtung an in Gefangenschaft gehaltenen Haussperlingen (** ***Passer domesticus*** **)**

Die Schnabelfärbung ist ein Merkmal, das sich bereits in kurzer Zeit stark verändern kann. Wir haben in einer Volierenpopulation weiblicher Haussperlingen (*Passer domesticus*) starke interindividuelle und saisonale Unterschiede in der Schnabelfarbe beobachtet und die Variation dieses Merkmals über den Brutzyklus hinweg anhand einfacher Kategorien beschrieben: i) das Vorhandensein oder Fehlen gelber Flecken auf dem Oberschnabel sowie ii) die Färbung des Schnabels in Bezug auf dessen Gesamterscheinung (hell/dunkel). Im Frühling hatten Weibchen entweder helle, hornfarbene, oder dunkle, nahezu schwarze Schnäbel, und bei etwa der Hälfte der Tiere stellten wir gelbe Flecken am Oberschnabel fest. Dies ändert sich jedoch deutlich nach dem Beginn der Brutzeit. Nach der ersten Brut wurde der Anteil an Individuen mit gelben Flecken am Schnabel geringer, und bei keiner der erfolgreich brütenden Weibchen war dieses Merkmal vorhanden. Während der nicht-reproduktiven Phase im Herbst wird die gelbe Pigmentierung des Oberschnabels jedoch bei allen Individuen wieder sichtbar. Außerdem nahm der Anteil der Weibchen mit hellen Schnäbeln zwischen den drei Messpunkten im Frühjar, Sommer und Herbst zu. Die ausgeprägten Unterschiede in der Schnabelfärbung zusammen mit den saisonalen Veränderungen deuten auf eine mögliche Signalwirkung hin und könnten eine Rolle in der sexuellen Selektion spielen.

## Introduction

Birds often develop colourful sexually selected features, with feathers being the best-studied examples. However, they are not the only aspect of the avian integument providing information to conspecifics. There are other traits signalling individual quality, such as the colouration of the bare areas of the skin (e.g., the legs, around the eyes and the neck) and the bill (Iverson and Karubian 2017). Unlike plumage colouration, which is determined during the moult and fades slowly over time, the pigmentation of the skin and bill can change rapidly in response to aspects of the individual condition (e.g., nutritional status) and between life history stages (Karubian et al. 2011; Rosenthal et al. 2012).

Most of our knowledge about bill colouration derives from studies on males, while the underlying mechanisms and the function of female bill colouration remain less understood. However, multiple observations indicate that this trait is not uniform across females but underlies notable interindividual variation in different species. Several studies showed that female bill colouration depends on the quality of the individual diet, stress, and hormonal changes (Pérez-Rodríguez and Viñuela 2008; Karubian et al. 2011; Rosenthal et al. 2012; Freitas et al. 2021; Romero-Diaz et al. 2022). In female Zebra finches (*Taeniopygia guttata*), for example, the redness of the bill is a sexually selected trait and more pigmented females have higher survival and reproductive success (Price and Burley 1994; Simons et al. 2012). Dey et al. (2015) showed in a systematic study that, carotenoid bill colouration is related to non-breeding sociality in passerines, suggesting a function in social signalling.

**Fig. 1:**
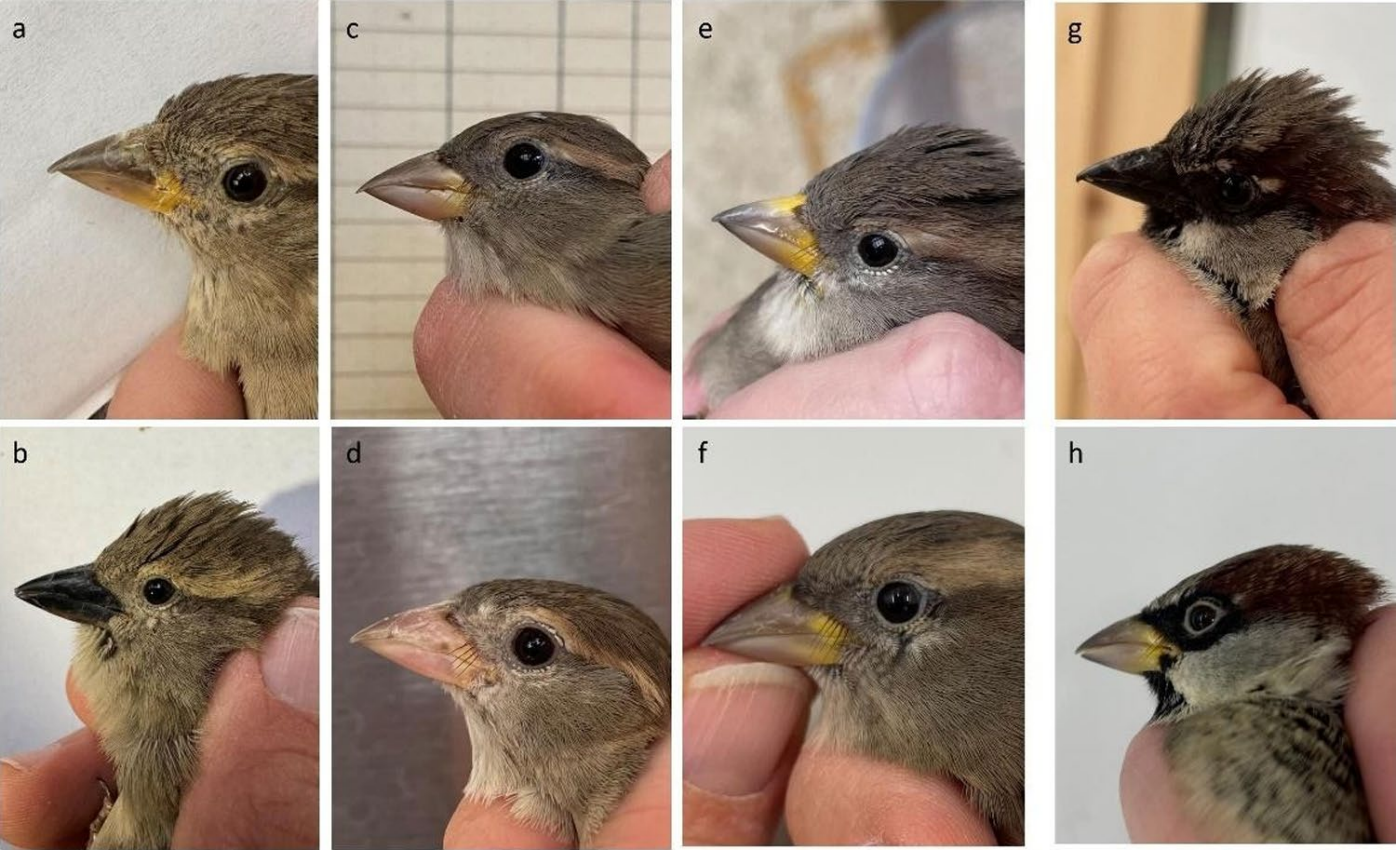
These pictures illustrate the variation of female bill colour over the season in comparison with males before the breeding season (g) and in the non-reproductive stage (h). Thereby **a** represents the typical appearance of females that were categorized as light and that displayed yellow colour on the upper mandible, **b** is the dark phenotype. **c** and **d** represent females that have just finished breeding and seem to display mainly pale colouration or loss of pigmentation. **e** and **f** both are examples of females in the post reproductive period, note the variation in the yellow and the similarity to the male (h)

During the preparations for an experiment on the effects of anthropogenic disturbance on the breeding strategy of captive House Sparrows (*Passer domesticus*), we noticed a strong phenotypic variation in female bill colouration. Individuals had either pale horn-coloured or dark brown, almost black, bills. Some females displayed yellow patches of varying intensity and size on their upper and lower mandibles (Fig. 1). The colouration of the male bill is well described and was shown to change with the reproductive status. Sexually regressed males display light brown bills with yellow markings, that darken during early spring in response to hormonal changes (Fig. 1) (Nichols 1935; Laucht et al. 2010). However, only one work mentions similar patterns in females. The study is detailed but limited to a few wild-caught individuals with varying and often unknown age and few re-captures (Nichols 1935). Hence, we here aimed to describe the two colouration traits in females, their change between seasons and a possible relationship with reproductive outcome.

## Methods

The study population consists of the first generation of House Sparrows bred from wild-caught individuals. The birds are individually marked and, between February and April, housed in large outdoor aviaries in same-sex flocks. Before transferring 60 males and 60 females into breeding facilities, the differences in the bill colouration traits were recorded. We refer to this time-point as *pre-breeding*. Another measurement was conducted *post-breeding*, when the offspring of the first clutch was independent and the females and fledgelings were separated from the males. In October, when the birds did not display any breeding behaviour, we recorded the bill colouration for the third time and refer to this time-point as *non-reproductive.*

We used simple categories to describe the two colouration traits of the bill: (i) pale horn-coloured bills were categorized as *light* and dark brown/black bills as *dark*; (ii) we noted the *presence* and *absence* of the conspicuous yellow pigmentation on the upper mandible (Fig. 1). We evaluated the categorizations of both traits by having two observers scoring 29 female birds in a blinded manner, resulting in only one disagreement. We used video recordings and regular monitoring of the nestboxes to assess the reproductive outcome. Although some females attempted a clutch, only individuals with at least one nestling were considered successful breeders.

All analyses were performed using R Studio v.4.4.1 (R Core Team 2024). We fitted a binomial mixed effects model with reproductive stage as a fixed effect and individual ID as a random intercept to analyse whether the colour traits change over the season (*n* = 168 observations from 56 individuals; “lme4” function, Bates et al. 2015). To assess the proportions and the relationship between the traits and breeding status of the females, chi-square tests were performed. Four females deceased during the breeding season and therefore were excluded from further analyses.

## Results

**Fig. 2:**
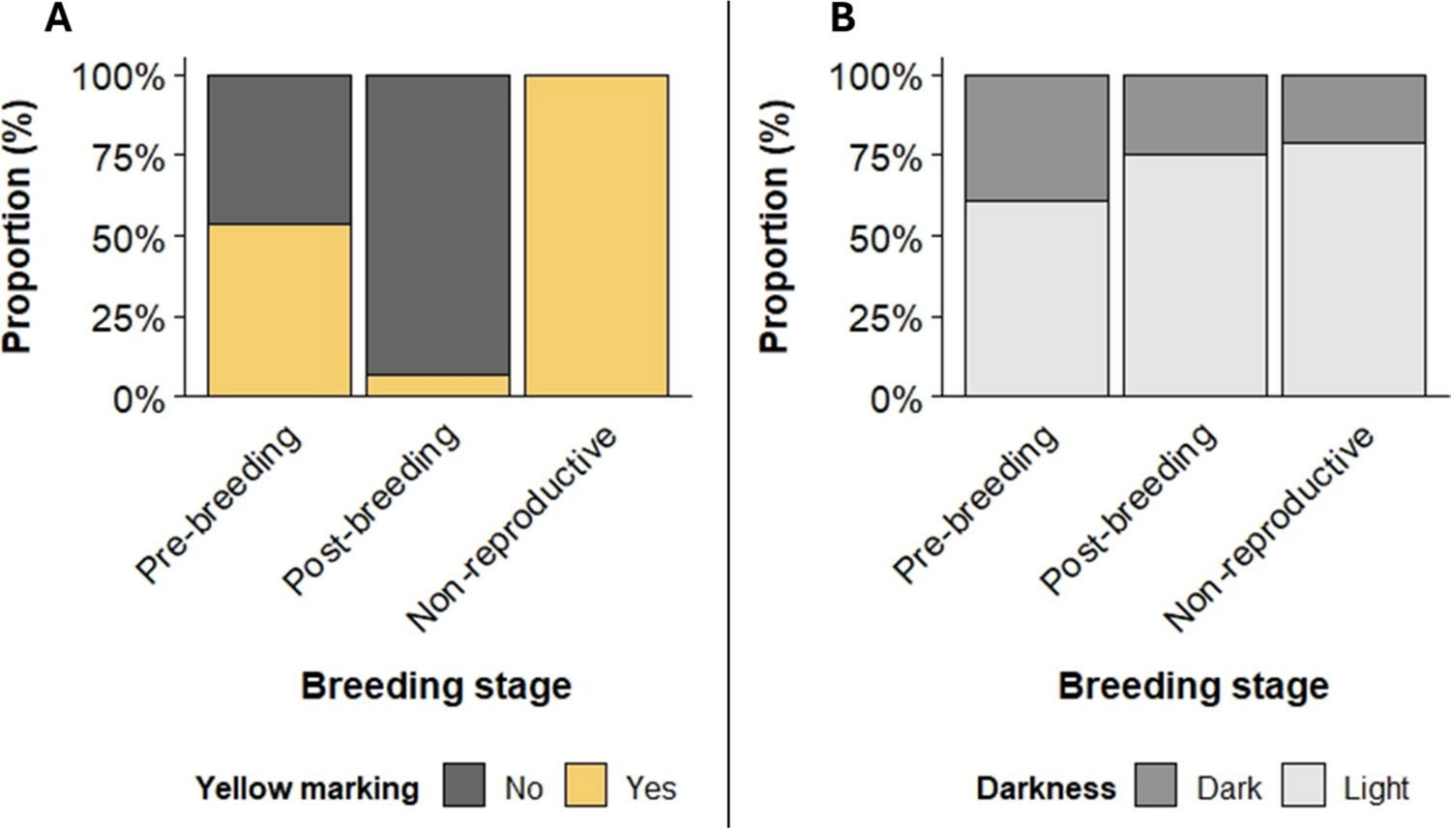
The figure A shows the change in the proportions of females with yellow markings on the upper mandible over the course of the three measurements corresponding to the pre-breeding, post-breeding and non-reproductive stage. The figure B illustrates the change of proportions of females with dark or light bills over the season

The odds of finding yellow markings on the base of the upper mandible decreased significantly in the post-breeding stage (pre-breeding:46.43%, post -breeding: 7.14%; Fig. 2A) (estimate ± SE = − 2.71 ± 5.84, *z* = − 4.64, *p* < 0.001). However, in October (non-reproductive sampling point) 100% of the birds exhibited yellow markings on the upper mandible (Fig. 2A).

Proportions calculated from the raw data showed an increase in the percentage of females with light bills across the three reproductive stages (pre-breeding: 60.71%, post -breeding: 75.00%, non-reproductive: 78.57%) (Fig. 2B). This was confirmed by the model showing that the odds of having a lighter bill later in the season increased. While there is only a trend for a significant difference between the pre- and post-breeding sampling point (*estimate* ± *SE* = 0.87 ± 0.48, *z* = 1.8, *p* = 0.07) a larger proportion of birds in the non-reproductive period has a light bill compared to pre-breeding (estimate ± SE = 1.12 ± 0.50, z = 2.24, *p* = 0.03).

Initially, females with light bills were more likely to have yellow pigmentation than dark-billed ones (*χ*^*2*^ = 11.89, *p* < 0.001; 22% of the dark females and 73% of light females). Surprisingly, neither darkness nor the yellow colour markings predicted a successful breeding attempt (yellow: χ^2^ = 0.46, *p* = 0.49, darkness: χ^2^ = 0.88, *p* = 0.35).

## Discussion

Our observations focused on two colour traits of the female bill across seasons and showed that both: (i) vary across different life history stages, (ii) appear highly variable between individuals, and (iii) that this effect appears to be independent of age as all females were yearlings.

The yellow marking on the upper mandible, was found in half of the females before breeding, became nearly absent during breeding (*post-breeding* measurement), and was present in all birds in the non-reproductive stage. This trait is likely a result of carotenoid-based pigmentation and might be an indicator of the female carotenoid status. Considering the tight link between carotenoids and the nutritional and health status of individuals, the depletion of bill colouration during the breeding season may be a consequence of nutrient allocation during reproduction (e.g., egg yolk, feeding) and potentially oxidative stress (Pérez-Rodríguez and Viñuela 2008; Freitas et al. 2021). Even birds that did not raise offspring lost the yellow markings, except for 4 individuals that were likely unpaired (video observations). These findings suggest that the stress load (e.g., social stress, maternal investment) arising over the breeding season, might contribute to the loss of pigmentation and that aspects of the yellow bill colouration, such as colour intensity or size of the pigmented area might indicate female condition (Pérez-Rodríguez and Viñuela 2008; Freitas et al. 2021).

Compared with the pre-breeding measurement, we observed more females with light bills after breeding and during the non-reproductive period and consequently fewer females with dark bills. We suggest that, as in males, the change in the pigmentation might coincide with the transition from the non-reproductive life history stage (October—February) to the breeding stage and is driven by variation in hormone levels (Laucht et al. 2010; Karubian et al. 2011). In male House Sparrows, dominance within the flock is related to the size of the black bib and variation in testosterone levels (Buchanan et al. 2010). There is evidence that also the darkness of the male bill is testosterone-dependent and a potential role in signalling testosterone-related behaviours, such as aggressiveness, has been discussed (Laucht et al. 2010). House Sparrows often breed in close vicinity and in females, the darkness of the bill may ultimately serve as an indicator of social dominance. This trait might be associated with advantages such as access to resources and nesting sites. Even though females sometimes display similar traits as males, their signalling function can differ. For example, melanin-based bill colouration in female, breeding Common Waxbills (*Estrilda astrild*) possibly serves in communication related to parental care (Cardoso and Batalha 2025). While the expression of some melanin-based ornaments is genetically determined (Roulin 2016), the question arises whether this also applies to the bill of female House Sparrows. Although some females were siblings, the sample size was too small to draw conclusions on the potential effects of relatedness on this trait.

We are aware that the simplified categorization of the colouration traits is a limitation in our study and a detailed description of the bill colouration requires the use of spectrometry. To better understand the role of female bill colouration, further studies including experimental procedures (e.g., carotenoid/hormone supplementation) and behavioural observations are necessary. Nevertheless, our approach was sufficient to reveal strong patterns suggesting a potential signalling function of female bill colouration in sexual or social selection in house sparrows encouraging further investigations.

## Data Availability

The data of this study are available from the corresponding author upon request.

## References

[CR1] Roulin A (2016) Condition-dependence, pleiotropy and the handicap principle of sexual selection in melanin-based colouration. Biol Rev Camb Philos Soc 91:328–348. 10.1111/brv.12171 25631160 10.1111/brv.12171

[CR2] Bates D, Mächler M, Bolker B, Walker S (2015) Fitting linear mixed-effects models using lme4. J Stat Softw 67(1):1–48. 10.18637/jss.v067.i01

[CR3] Buchanan KL, Evans MR, Roberts ML et al (2010) Does testosterone determine dominance in the house sparrow *Passer domesticus*? An experimental test. J Avian Biol 41:445–451. 10.1111/j.1600-048x.2010.04929.x

[CR4] Cardoso GC, Batalha HR (2025) A female-specific color signal? Black-mottled bills indicate breeding in female common waxbills. Am Nat 206:80–86. 10.1086/735832 40577830 10.1086/735832

[CR5] Dey CJ, Valcu M, Kempenaers B, Dale J (2015) Carotenoid-based bill coloration functions as a social, not sexual, signal in songbirds (Aves: Passeriformes). J Evol Biol 28:250–258. 10.1111/jeb.12560 25430614 10.1111/jeb.12560

[CR6] Freitas R, Marques C, Cardoso GC, Trigo S (2021) Ecological effects on female bill colour explain plastic sexual dichromatism in a mutually-ornamented bird. Sci Rep 11:14970. 10.1038/s41598-021-93897-z 34294752 10.1038/s41598-021-93897-z PMC8298529

[CR7] Iverson ENK, Karubian J (2017) The role of bare parts in avian signaling. Auk 134:587–611. 10.1642/auk-16-136.1

[CR8] Karubian J, Lindsay WR, Schwabl H, Webster MS (2011) Bill coloration, a flexible signal in a tropical passerine bird, is regulated by social environment and androgens. Anim Behav 81:795–800. 10.1016/j.anbehav.2011.01.012

[CR9] Laucht S, Kempenaers B, Dale J (2010) Bill color, not badge size, indicates testosterone-related information in house sparrows. Behav Ecol Sociobiol 64:1461–1471. 10.1007/s00265-010-0961-9 20730125 10.1007/s00265-010-0961-9 PMC2920409

[CR10] Nichols JT (1935) Seasonal and individual variations in house sparrows. Bird-Band 6:11. 10.2307/4509324

[CR11] Pérez-Rodríguez L, Viñuela J (2008) Carotenoid-based bill and eye ring coloration as honest signals of condition: an experimental test in the red-legged partridge (*Alectoris rufa*). Naturwissenschaften 95:821–830. 10.1007/s00114-008-0389-5 18470503 10.1007/s00114-008-0389-5

[CR12] Price DK, Burley NT (1994) Constraints on the evolution of attractive traits: selection in male and female zebra finches. Am Nat 144:908–934. 10.1086/285718

[CR13] R Core Team (2024) R: A Language and Environment for Statistical Computing.

[CR14] Romero-Diaz C, Silva PA, Soares MC et al (2022) Oestradiol reduces female bill colour in a mutually ornamented bird. Proc Biol Sci 289:20221677. 10.1098/rspb.2022.1677 36476006 10.1098/rspb.2022.1677 PMC9554724

[CR15] Rosenthal MF, Murphy TG, Tarvin N, Darling KA (2012) Ornamental bill color rapidly signals changing condition. J Avian Biol 43:553–564. 10.1111/j.1600-048X.2012.05774.x

[CR16] Simons MJP, Briga M, Koetsier E et al (2012) Bill redness is positively associated with reproduction and survival in male and female zebra finches. PLoS ONE 7:e40721. 10.1371/journal.pone.0040721 22808243 10.1371/journal.pone.0040721 PMC3395645

